# Diagnostic performance of commercial AI systems versus participating radiologists for pulmonary nodule detection in routine clinical practice

**DOI:** 10.1007/s11604-026-02029-y

**Published:** 2026-06-18

**Authors:** Hiroaki Watanabe, Hiroyuki Morisaka, Kaori Aoyagi, Ryota Tozuka, Tihan Wumu, Takanori Ii, Kasane Sakamoto, Tomomi Yato, Shintaro Ichikawa

**Affiliations:** 1 Department of Diagnostic Radiology, University of Yamanashi, 1110, Shimokato, Chuo, Yamanashi, 409-3898 Japan; 2 Department of Therapeutic Radiology, University of Yamanashi, Chuo, Yamanashi, Japan; 3 Department of Diagnostic Radiology, Yamanashi Central Hospital, Kofu, Yamanashi, Japan; 4 Division of Radiology, University of Yamanashi Hospital, Chuo, Yamanashi, Japan

**Keywords:** Pulmonary nodule, Lung cancer, Artificial intelligence, Computed tomography, Observer variation

## Abstract

**Purpose:**

To evaluate the diagnostic performance of two commercial artificial intelligence (AI) systems versus that of radiologists on routine clinical chest computed tomography (CT) and to identify the imaging characteristics that limit AI performance.

**Materials and Methods:**

We retrospectively analyzed the 5-mm-slice chest CT of 102 patients (353 nodules or masses). The detection performance of two board-certified radiologists and two commercial AI systems was compared against an expert-established reference standard. Sensitivity, false positives (FPs) per scan, and positive predictive value (PPV) were evaluated. Logistic regression identified factors influencing detection.

**Results:**

The radiologists demonstrated higher sensitivity (90.4–94.3%) than the AI systems (80.2–89.2%) and significantly fewer FPs per scan (1.61–3.56 vs. 5.28–7.97; *p* < 0.001), resulting in superior PPVs. Multivariate analysis revealed divergent limitations: radiologists were challenged by intrinsic features (e.g., ground-glass nodules [GGNs]), whereas AI performance was degraded by case-level complexity (multiple nodules), specific locations (central), and atypical morphologies (large masses).

**Conclusion:**

In this study, the participating radiologists outperformed the commercial AI systems in routine clinical settings. Both exhibited distinct weakness profiles. Current AI systems are best suited as complementary tools rather than autonomous readers, provided FPs are managed effectively.

**Supplementary Information:**

The online version contains supplementary material available at https://doi.org/10.1007/s11604-026-02029-y.

## Introduction

Early detection of lung cancer via low-dose computed tomography (LDCT) significantly reduces the risk of mortality, leading to the global implementation of screening programs and management guidelines [1–6]. However, nodule detection is equally critical in daily clinical practice, where “opportunistic screening” on standard diagnostic computed tomography (CT) frequently identifies incidental findings. The surging volume of these examinations has intensified radiologist workloads, raising concerns about diagnostic errors, particularly when interpreting complex clinical scans characterized by variable image quality and co-existing pathologies.

To address these challenges, computer-aided detection systems using artificial intelligence (AI) were developed to assist radiologists across various imaging protocols [4, 7]. Although these AI systems often improve detection sensitivity, this benefit is frequently accompanied by a reduction in specificity, resulting in a high rate of false-positive (FP) findings [8, 9]. Furthermore, their application to routine clinical imaging presents unique challenges. Unlike screening datasets, which typically consist of standardized thin-slice LDCT images, real-world clinical datasets often involve thicker slices, variable radiation doses, and the presence of complex comorbid findings such as consolidation or pleural effusions. AI performance validated on screening data may not directly translate to this heterogeneous clinical environment. Although deep learning models demonstrate high performance on thin-slice or ultra-high-resolution images [10], they were shown to exhibit suboptimal performance when applied to thick-slice scans [11]. Additionally, a systematic review revealed that many AI algorithms perform worse on external validation datasets compared to their development data, highlighting a critical need for validation in real-world settings [8, 12]. This combination of a high FP rate and uncertain real-world performance remains a significant barrier to clinical adoption.

Therefore, we aimed to conduct a direct performance comparison of two commercially available AI systems versus board-certified radiologists using a dataset derived specifically from routine clinical practice. The primary objective was to evaluate detection sensitivity and positive predictive value (PPV) for pulmonary nodules or masses and lung cancers on clinical chest CT scans. A secondary objective was to identify specific imaging characteristics—such as nodule or mass type or co-existing lung pathology—that differentially affect human versus AI performance in this setting. By clarifying the capabilities and limitations of AI tools within the context of daily diagnostic radiology, this research seeks to provide essential insights for their effective integration into clinical workflows.

## Materials and methods

### Study design and ethical considerations

This was a retrospective, single-center, non-interventional observational study. The research protocol was approved by the institutional research ethics board of the University of Yamanashi (Approval No. 2982), and the requirement for individual informed consent was waived given the retrospective nature of the study.

We retrospectively identified patients who underwent chest CT at our institution from April 7, 2023, to October 20, 2023. From this cohort, 102 cases wherein lung cancer was either pathologically confirmed or clinically suspected, including cases with pulmonary metastases, were selected. For each selected case, chest CT image data acquired with a 5-mm slice thickness and reconstructed with a lung kernel were collected. Additionally, the following clinical information was retrieved from patient records: sex, age, body mass index (BMI), smoking history, history of malignant tumors, family history, presence or absence of chronic lung disease, and presence or absence of an immunosuppressive state. Chronic lung disease was defined primarily as the presence of chronic obstructive pulmonary disease (COPD) or interstitial lung disease (ILD). An immunosuppressive state was defined as meeting any of the following criteria: (1) use of immunosuppressants or systemic corticosteroids (equivalent to ≥ 10 mg/day of prednisolone) for 2 weeks or longer; (2) currently receipt or receipt of chemotherapy within the past 6 months; (3) human immunodeficiency virus infection with a CD4 count < 200/μL or other overt immunodeficiency disorders; or (4) a history of solid organ or hematopoietic stem cell transplantation with ongoing immunosuppressive therapy.

### CT imaging protocol and reconstruction

The CT scans were performed using the following multi-detector CT scanners: Aquilion ONE, Aquilion ONE / PRISM Edition, or Aquilion Precision (Canon Medical Systems, Otawara, Japan). Detailed scanning and reconstruction parameters for each scanner are summarized in Table 1. Efforts were made to standardize acquisition protocols across different devices to minimize variability in image quality. Automatic exposure control (AEC) was used for all acquisitions, with target standard deviations (at 5 mm slice thickness) set to 12 for the Aquilion ONE, 16 for the PRISM Edition, and 17 for the Precision. The mean volume CT dose index (CTDIvol) and dose-length product (DLP) for the study cohort were 13.2 ± 8.5 mGy and 777.1 ± 487.2 mGy·cm, respectively.

**Table 1:** CT scanning and reconstruction parameters

Category	Parameter	Scanning parameters
Acquisition	Tube voltage (kVp)	120
	Tube current (mA)	Auto exposure control (ONE)
		Auto exposure control with Volume EC (PRISM Edition, Precision)
	AEC target (HU)*	12 (ONE)
		16 (PRISM Edition)
		17 (Precision)
	Rotation time (s)	0.45†
	Beam pitch	0.813 (ONE)
		1.388 (PRISM Edition, Precision)
Reconstruction	Reconstructed section thickness (mm)	5
	Reconstructed section interval (mm)	5
	Reconstruction kernel	AIDR 3D Weak (ONE)
		AiCE Lung Mild (PRISM Edition, Precision)
	Matrix size	512 × 512
	Deep learning reconstruction	None (ONE)
		AiCE** (PRISM Edition, Precision)
Radiation dose	CTDIvol (mGy)	13.2 ± 8.5 (3.3–48.0)
	DLP (mGy·cm)	777.1 ± 487.2 (145.1–3104.4)

^*^Target standard deviation (SD) was defined at a 5-mm slice thickness. † Rotation time was adjusted (range: 0.275–0.45 s) for patients unable to hold their breath. ** AiCE: Advanced Intelligent Clear-IQ Engine. ONE = Aquilion ONE; PRISM Edition = Aquilion ONE / PRISM Edition; Precision = Aquilion Precision

AEC: automatic exposure control, AIDR 3D: Adaptive Iterative Dose Reduction 3D, CT: computed tomography, CTDIvol: volume CT dose index, DLP: dose-length product

### Reference standard

To establish a robust and unbiased reference standard, an initial evaluation was performed by a board-certified thoracic radiologist with 10 years of experience (Reference Evaluator 1). Subsequently, a second, highly experienced board-certified radiologist with 19 years of experience (Reference Evaluator 2) independently reviewed these initial findings comprehensively, referencing all available clinical/pathological reports, as well as the detection results from the two human readers (Readers A and B) and both AI systems. Any discrepancies regarding the presence or characteristics of a nodule were resolved through consensus discussion between Reference Evaluators 1 and 2. During this rigorous review process, 13 lesions were identified as pleural nodules rather than true intrapulmonary nodules and were excluded from the analysis. Ultimately, a total of 353 true pulmonary nodules or masses were included in the final reference standard. For each confirmed nodule or mass, the evaluators documented the following imaging characteristics: nodule size (classified into < 6 mm, 6–8 mm, and > 8 mm), presence of a mass (≥ 30 mm), location (central or peripheral), internal structure (ground-glass or solid), adjacency to the pleura (juxta-pleural), adjacency to blood vessels (juxta-vascular), presence of consolidation, presence of multiple nodules (≥ 10), and presence of an atypical cyst. To ensure a fair baseline and align with the approved detection specifications of AI 1, the minimum size threshold for inclusion in the reference standard was set at ≥ 3 mm for solid nodules and ≥ 5 mm for ground-glass nodules (GGNs). Furthermore, because this study utilized 5 mm slice thickness, precise subclassification of nodules with ground-glass opacity (e.g., pure ground-glass vs. part-solid) is technically limited because of the partial volume effect. Therefore, these were collectively classified as GGNs.

### Image interpretation by radiologists and AI systems

The CT datasets were independently interpreted by two board-certified radiologists (Reader A and Reader B) and the two commercial AI systems. The human readers (A and B) were blinded to all clinical information and to each other’s findings. Reader A and Reader B had 19 and 5 years of experience in thoracic radiology, respectively. The human readers were instructed to detect and annotate all pulmonary nodules or masses that met the predefined size criteria (≥ 3 mm for solid, ≥ 5 mm for GGNs). To evaluate pure detection performance and ensure a fair comparison with the AI systems, the readers were instructed not to filter their findings based on clinical management guidelines (e.g., the Fleischner Society criteria). Their interpretations, along with the AI results, were subsequently used to establish the reference standard. Their task was solely to detect and annotate all locations they identified as pulmonary nodules or masses.

Concurrently, the same datasets were analyzed by two commercially available AI-based lung nodule/mass detection systems. The first system was the Lung Nodule Detection program (FS-AI688) integrated into the SYNAPSE SAI viewer (Fujifilm Corporation, Tokyo, Japan; hereafter referred to as AI 1). The second system was Plus.Lung.Nodule version 2.2.0 (Plusman LLC, Tokyo, Japan; hereafter referred to as AI 2), a dedicated chest CT AI software. These two AI systems were selected because they represent widely implemented, commercially available, and regulatory-approved tools for chest CT in Japan. According to the manufacturers’ package inserts, AI 1 utilizes different size thresholds based on nodule characteristics (≥ 3 mm for solid nodules and ≥ 5 mm for GGNs), which we adopted for our reference standard. AI 2 operates with a default detection threshold of ≥ 2 mm for all nodule types. Both systems were operated according to these standard clinical configurations.

### Outcome measures and variables

The primary outcome measures for this study were detection sensitivity, PPV, and the mean number of FPs per scan. The secondary objective was to identify which imaging characteristics of nodules or masses influence detection performance. For this analysis, the following variables, as documented for each nodule or mass in the reference standard, were evaluated as potential predictors: nodule/mass size (classified as < 6 mm, 6–8 mm, > 8 mm), size ≥ 30 mm, lung lobe location, location (peripheral or central), shape (polygonal or round), border definition (clear or unclear), internal characteristics (solid or GGN), presence of an atypical cyst, presence of a nodule or mass along a cyst wall, proximity to pleura or blood vessels, presence of consolidation, and presence of 10 or more multiple nodules or masses.

### Statistical analysis

Comparisons of sensitivity among the four groups (Reader A, Reader B, AI 1, and AI 2) were performed using Cochran’s Q test, followed by post-hoc McNemar’s tests with Holm–Bonferroni correction for multiple comparisons. The mean number of FPs per scan was compared using the Friedman test, followed by post-hoc Wilcoxon signed-rank tests with Holm–Bonferroni correction. For PPV, 95% confidence intervals (CIs) were calculated using the Wilson score method, and comparisons were assessed based on the overlap of CIs. Finally, to identify imaging characteristics that influence detection performance, we performed a two-step analysis. First, univariate logistic regression analyses were conducted for each variable to calculate crude odds ratios (ORs). For variables where complete separation occurred (i.e., zero false negatives or FPs), Fisher’s exact test was applied. Second, variables with a *p*-value < 0.20 in the univariate analysis were selected and included in the multivariate logistic regression model to determine independent predictors of detection. Additionally, to visualize the divergence in detection profiles across all detectors, seven characteristic features were selected based on the multivariate analysis results. For these specific features, crude ORs from the univariate analysis were plotted in a forest plot to ensure data availability for comparison across all four detectors.

## Results

### Patient and nodule or mass characteristics

We included 102 patients in the study. The demographic and clinical characteristics of the cohort are presented in Table 2. The patients’ mean age was 74.9 ± 8.5 years, and the mean BMI was 22.5 ± 2.7. Key clinical histories included prior lung cancer in 12 patients (11.8%) and other malignancies in 50 patients (49.0%).

**Table 2:** Patient demographics and nodule/mass characteristics

Characteristic	Value
Patient level	(n = 102)
Age (years), mean ± SD	74.9 ± 8.5
Sex, n (%)	
Male	68 (66.7)
Female	34 (33.3)
Body mass index (kg/m^2^), mean ± SD	22.5 ± 2.7
Smoking history, n (%)	73 (71.6)
Clinical history, n (%)	
Prior lung cancer	12 (11.8)
Other malignancies	50 (49.0)
Family history of non-lung malignancies	22 (28.9)
Chronic lung disease	20 (19.6)
Immunosuppressive state*	8 (7.8)
Nodule/Mass level (reference standard)	(n = 353)
Size and type, n (%)	
Solid (n = 319, 90.4%)	
< 6 mm	90 (25.5)
6–8 mm	83 (23.5)
> 8 mm	146 (41.4)
GGN (n = 34, 9.6%)	
< 6 mm	6 (1.7)
6–8 mm	4 (1.1)
> 8 mm	24 (6.8)
Location, n (%)	
Peripheral	254 (72.0)
Central	99 (28.0)
Morphological and surrounding features, n (%)	
Mass (≥ 30 mm)	20 (5.7)
Juxta-pleural	127 (36.0)
Juxta-vascular	35 (9.9)
Consolidation	8 (2.3)
Atypical cyst	7 (2.0)
Case complexity, n (%)	
Multiple nodules (≥ 10 per case)	138 (39.1)

Values are presented as mean ± standard deviation (SD) or number (percentage). * An immunosuppressive state was defined as meeting any of the following criteria: (1) use of immunosuppressants or systemic corticosteroids (≥ 10 mg/day of prednisolone) for ≥ 2 weeks; (2) chemotherapy within the past 6 months; (3) human immunodeficiency virus infection (CD4 < 200/μL) or other immunodeficiency; or (4) a history of solid organ or hematopoietic stem cell transplantation

SD: standard deviation, GGN: ground-glass nodule

The reference standard identified a total of 353 pulmonary nodules or masses across these patients. This sample size provided sufficient statistical power (> 80%) to detect a 10% difference in sensitivity between groups, with a two-sided significance level (α) of 0.05. The imaging characteristics of the nodules are summarized in Table 2. Regarding size, 96 nodules or masses (27.2%) were classified as < 6 mm in diameter (with lower bounds of 3 mm for solid nodules and 5 mm for GGNs), 87 (24.6%) were 6–8 mm, and 170 (48.2%) were > 8 mm. Solid nodules or masses were the dominant type, accounting for 319 of the 353 nodules (90.4%), followed by GGNs (n = 34, 9.6%). The majority of nodules or masses were located in the peripheral lung (n = 254, 72.0%). Other morphological and surrounding features included masses ≥ 30 mm (n = 20, 5.7%), juxta-pleural location (n = 127, 36.0%), juxta-vascular location (n = 35, 9.9%), association with consolidation (n = 8, 2.3%), and atypical cysts (n = 7, 2.0%). Additionally, regarding case complexity, 138 nodules (39.1%) were identified in cases with a high nodule burden (≥ 10 nodules per case), corresponding to six patients (5.9% of the study cohort).

### Nodule or mass detection sensitivity and FPs

**Fig. 1:**
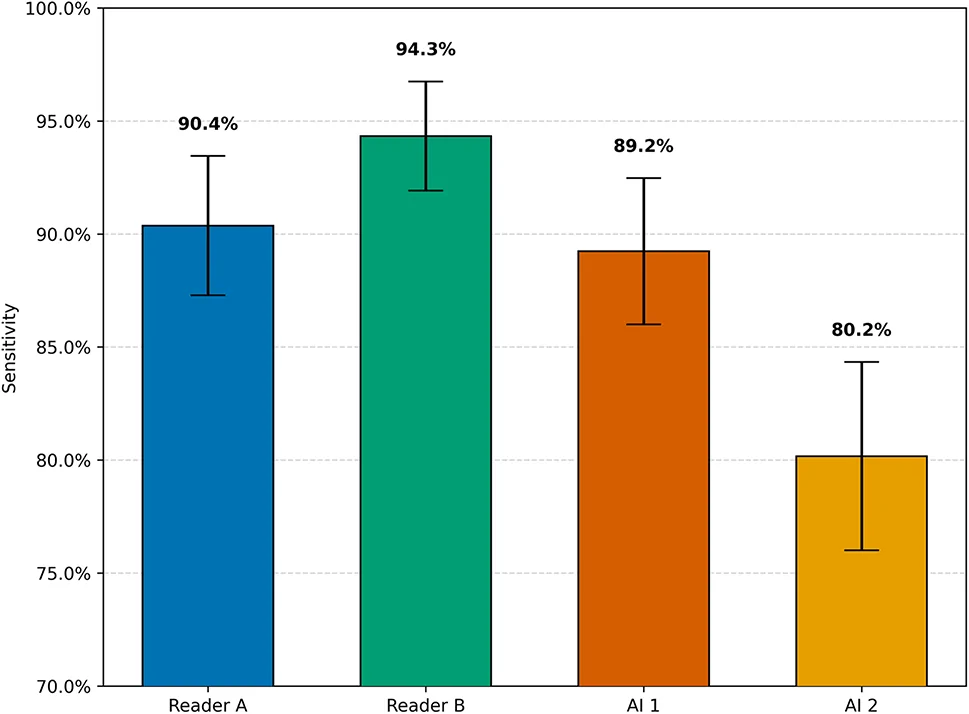
Comparison of overall nodule or mass detection sensitivity among radiologists (Reader A and Reader B) and artificial intelligence (AI) systems (AI 1 and AI 2). Error bars represent 95% confidence intervals. AI 1 = Fujifilm Corporation; AI 2 = Plusman LLC

The overall detection sensitivity for each reader and AI system is presented in Fig. 1. Reader B demonstrated the highest sensitivity at 94.3% (95% CI: 91.4–96.3%), followed by Reader A at 90.4% (95% CI: 86.8–93.0%). The sensitivities for the AI systems were 89.2% for AI 1 (95% CI: 85.6–92.1%) and 80.2% for AI 2 (95% CI: 75.7–84.0%). There was a statistically significant difference in sensitivity among the four groups (*p* < 0.001, Cochran’s Q test). Post-hoc pairwise comparisons using McNemar’s test with Holm–Bonferroni correction revealed that Reader B’s sensitivity was significantly higher than that of Reader A, AI 1, and AI 2.

**Fig. 2:**
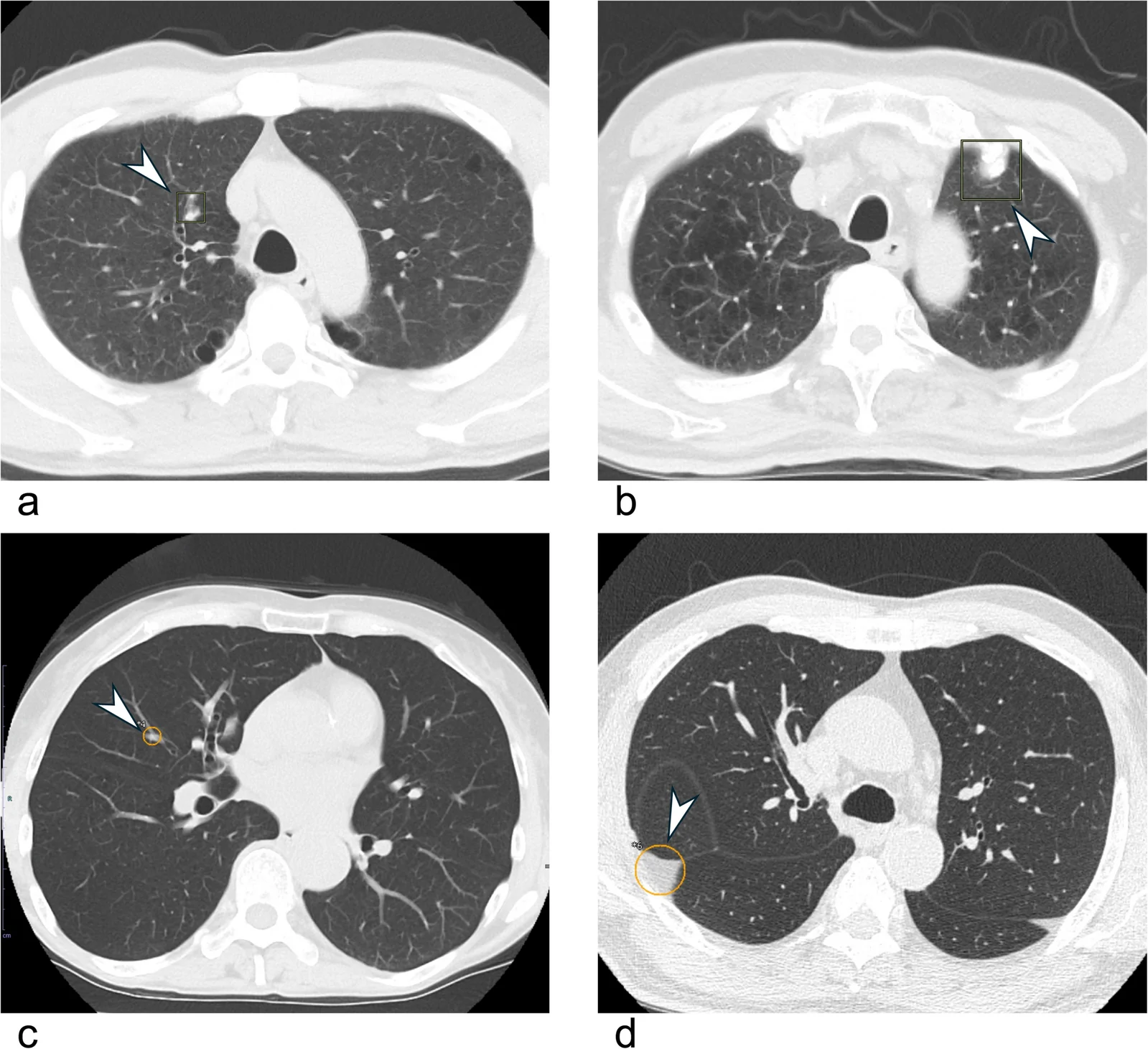
Examples of false-positive findings generated by artificial intelligence (AI) systems. The AI systems occasionally misidentified normal anatomical structures or benign changes as nodules. The false-positive detections seen here correspond to: **a** a vessel bifurcation; **b** a sternocostal joint; **c** another vessel bifurcation; and **d** interlobar pleural effusion mimicking a nodule shape

The mean number of FPs per scan was 1.61 for Reader A, 3.56 for Reader B, 5.28 for AI 1, and 7.97 for AI 2. The Friedman test indicated a significant difference in the number of FPs per scan across the groups (*p* < 0.001). Post-hoc analysis using Wilcoxon signed-rank tests with Holm–Bonferroni correction showed that both AI systems produced significantly more FPs per scan than the human readers (adjusted *p* < 0.001 for all comparisons). Furthermore, AI 2 had significantly more FPs than AI 1 (adjusted *p* < 0.001). Common sources of FP findings generated by the AI systems included normal anatomical structures and benign physiological changes, such as vessel bifurcations and osteo-cartilaginous structures (Fig. 2).

**Fig. 3:**
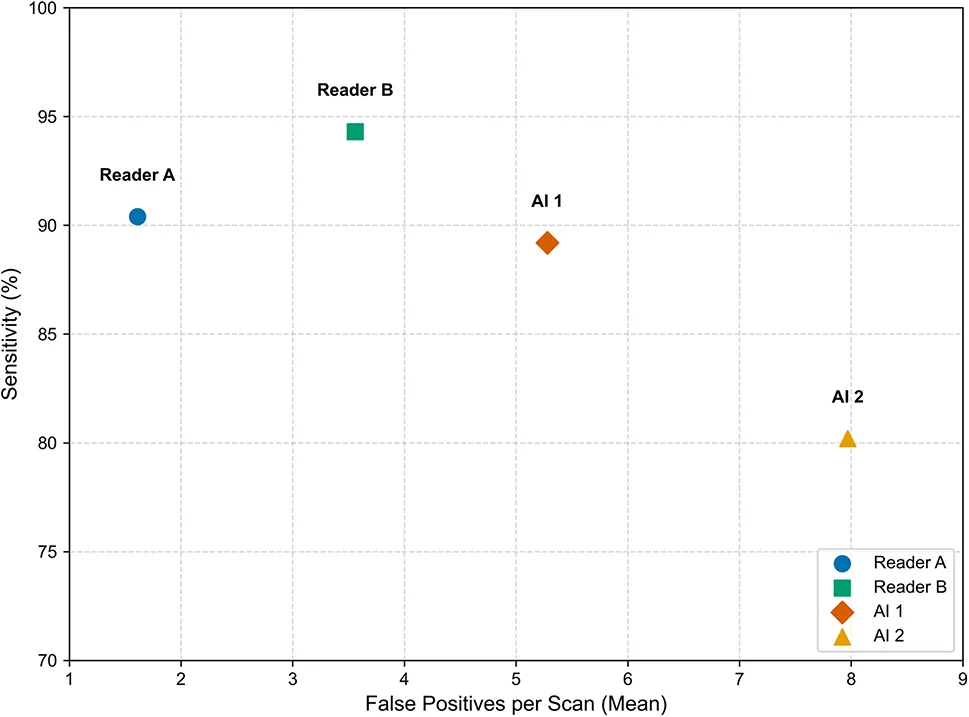
Sensitivity versus false positives per scan for each detector. The plot demonstrates the trade-off between detection capability and false-positive rate. Radiologists (circles and squares) are located toward the left (lower false positives), whereas AI systems (diamonds and triangles) are located toward the right. AI 1 = Fujifilm Corporation; AI 2 = Plusman LLC. Abbreviations: GGN: ground-glass nodule

PPV was higher for the human readers (Reader A: 66.0% [95% CI: 61.7–70.1%], Reader B: 47.8% [95% CI: 44.2–51.6%]) than for the AI systems (AI 1: 36.9% [95% CI: 33.7–40.2%]; AI 2: 25.8% [95% CI: 23.3–28.5%]). The lack of overlap in the 95% CIs indicated that Reader A’s PPV was significantly higher than that of Reader B and both AI systems. Similarly, AI 1 demonstrated a significantly higher PPV than AI 2. The overall diagnostic performance is summarized in Table 3, and the relationship between sensitivity and FPs is visualized in Fig. 3.

**Table 3:** Diagnostic performance comparison

Metric	Reader A	Reader B	AI 1	AI 2
Sensitivity (%) [95% CI]	90.4 (86.8–93.0)	94.3 (91.4–96.3)	89.2 (85.6–92.1)	80.2 (75.7–84.0)
Mean false positives per scan	1.61	3.56	5.28	7.97
PPV (%), [95% CI]	66.0 (61.7–70.1)	47.8 (44.2–51.6)	36.9 (33.7–40.2)	25.8 (23.3–28.5)

AI 1 = Fujifilm Corporation; AI 2 = Plusman LLC

CI: confidence interval, PPV: positive predictive value

### Factors influencing detection performance

**Fig. 4:**
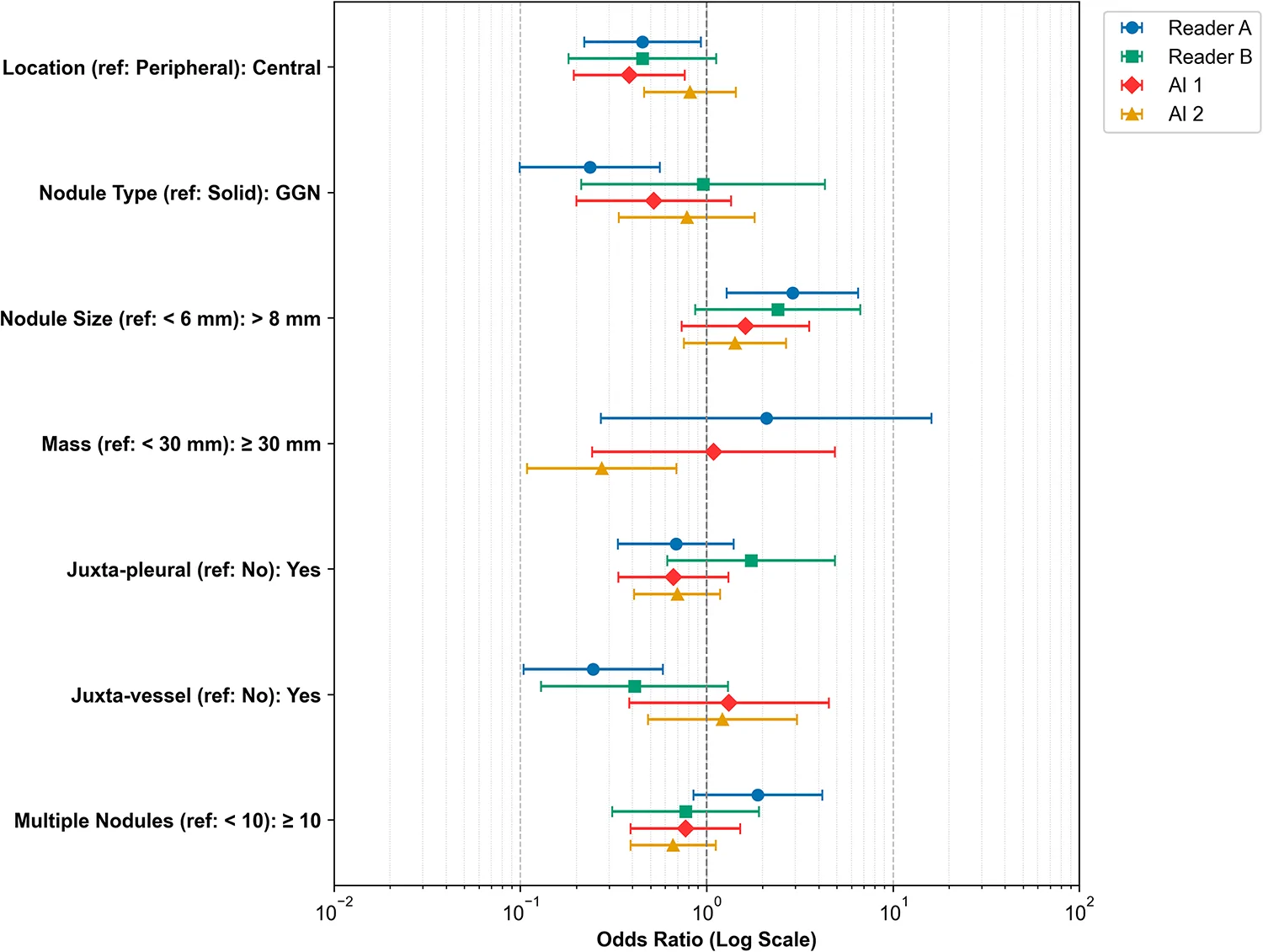
Forest plot of crude odds ratios (univariate analysis) for seven selected factors influencing nodule or mass detection. An odds ratio < 1 (left of the dashed vertical line) indicates characteristics associated with lower detection sensitivity. The plot highlights the divergence in limitations: human readers (Reader A, blue) are primarily challenged by intrinsic nodule features, such as ground-glass opacity and juxta-vascular location, whereas AI systems (AI 1, red; AI 2, orange) are more susceptible to central location, complex case features (multiple nodules), and atypical morphologies (large masses). Error bars represent 95% confidence intervals. *Note:* To allow comparison across all detectors, this plot displays univariate odds ratios, unlike Table 4, which presents multivariate results. Data for Reader B in the "Mass (≥ 3 cm)" category are not displayed because the odds ratio could not be calculated due to perfect detection (100% sensitivity) for this feature. AI 1 = Fujifilm Corporation; AI 2 = Plusman LLC

**Fig. 5:**
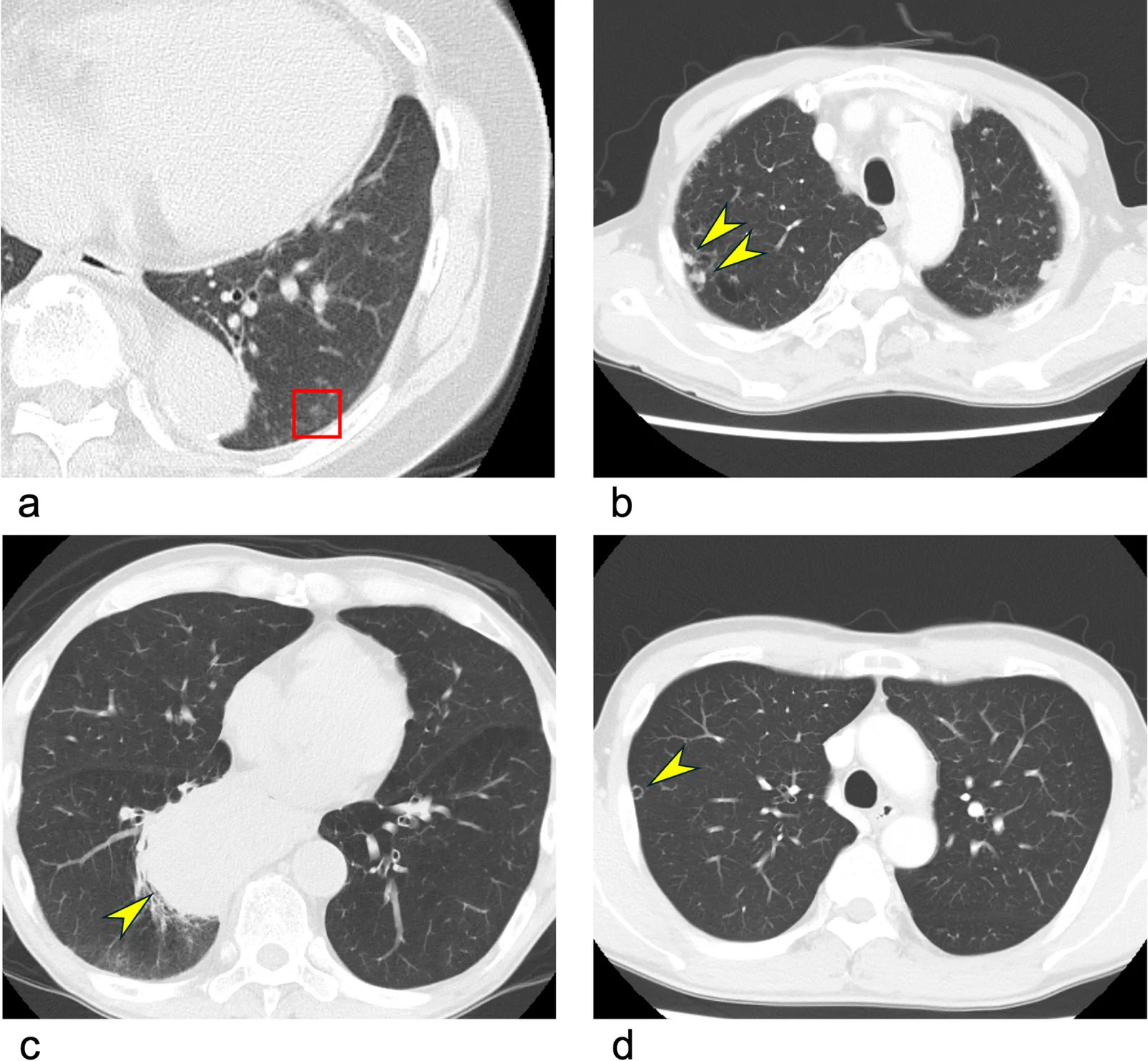
Representative cases illustrating divergent detection patterns between radiologists and AI. **a** A pure ground-glass nodule (GGN) detected by both AI systems but missed by both board-certified radiologists. This illustrates the AI’s sensitivity to subtle density differences. **b** A case with multiple nodules. Both radiologists correctly identified the nodules, whereas both AI systems failed to detect them, likely because of the complexity of the high nodule burden. **c** A large lung mass. Despite its conspicuity, this lesion was detected by both radiologists but missed by both AI systems. **d** A cystic nodule (lung cancer associated with cystic airspaces). Both radiologists detected this atypical lesion, whereas both AI systems failed to identify it as a nodule.

To identify specific imaging characteristics affecting detection, we first performed univariate logistic regression analyses (or Fisher’s exact tests for variables with complete separation), followed by multivariate logistic regression for variables with a *p*-value < 0.20 (Table 4, Supplemental Table 1). For Reader A, intrinsic nodule characteristics were the primary determinants of performance. Larger nodule size (> 8 mm) was a significant independent predictor of successful detection (adjusted OR [aOR]: 5.72 [95% CI: 2.17–15.08], *p* < 0.001). Conversely, detection was significantly lower for GGNs than for solid nodules (aOR: 0.21 [95% CI: 0.07–0.61], *p* = 0.004), as well as for nodules located centrally (aOR: 0.40 [95% CI: 0.18–0.87], *p* = 0.022), adjacent to vessels (aOR: 0.30 [95% CI: 0.11–0.80], *p* = 0.016), or associated with consolidation (aOR: 0.07 [95% CI: 0.01–0.41], *p* = 0.004). Reader B, who achieved the highest overall sensitivity, exhibited no statistically significant performance decrement associated with any specific imaging characteristic in the multivariate analysis, although trends were observed for centrally located nodules (aOR: 0.45 [95% CI: 0.18–1.13], *p* = 0.089) and juxta-vascular nodules (aOR: 0.41 [95% CI: 0.12–1.37], *p* = 0.149). In contrast, the AI systems showed distinct limitations. AI 1 performance was significantly affected by nodule location, demonstrating an independent weakness for centrally located nodules (aOR: 0.35 [95% CI: 0.18–0.71], *p* = 0.004). AI 2, however, was highly susceptible to broader case-level factors and atypical morphologies. The presence of multiple nodules (≥ 10 per case) was an independent predictor of lower detection for AI 2 (aOR: 0.54 [95% CI: 0.31–0.95], *p* = 0.033). Furthermore, AI 2 exhibited a significant weakness for masses ≥ 30 mm, which were associated with substantially lower detection (aOR: 0.24 [95% CI: 0.09–0.64], *p* = 0.004). To directly compare the weakness profiles across all detectors, Fig. 4 presents the crude ORs (univariate analysis) for seven key factors identified as significant in the multivariate analysis. Representative cases illustrating these divergent detection patterns are shown in Fig. 5. Specifically, the figure displays subtle GGNs missed by radiologists but detected by AI and, conversely, complex or atypical presentations missed by AI but detected by radiologists.

**Table 4:** Factors influencing detection performance (univariate and multivariate analysis)

Detector	Factor	Category	Univariate OR (95% CI)	*p*-value	Multivariate aOR (95% CI)	*p*-value
Reader A	Nodule size	< 6 mm	Reference		Reference	
		6–8 mm	2.29 (0.89–5.86)	0.085	2.28 (0.80–6.50)	0.123
		> 8 mm	2.89 (1.28–6.52)	0.011	5.72 (2.17–15.08)	< 0.001
	Location	Peripheral	Reference		Reference	
		Central	0.45 (0.22–0.93)	0.031	0.40 (0.18–0.87)	0.022
	Internal structure	Solid	Reference		Reference	
		GGN	0.24 (0.10–0.56)	0.001	0.21 (0.07–0.61)	0.004
	Juxta-vascular	Yes	0.25 (0.10–0.58)	0.001	0.30 (0.11–0.80)	0.016
	Consolidation	Yes	0.16 (0.04–0.72)	0.017	0.07 (0.01–0.41)	0.004
	Multiple nodules	Yes (≥ 10)	1.89 (0.85–4.17)	0.117	1.18 (0.47–2.96)	0.723
Reader B	Nodule size	< 6 mm	Reference		Reference	
		6–8 mm	2.15 (0.64–7.24)	0.218	2.03 (0.59–6.95)	0.261
		> 8 mm	2.41 (0.87–6.69)	0.092	2.67 (0.95–7.53)	0.064
	Location	Peripheral	Reference		Reference	
		Central	0.45 (0.18–1.13)	0.089	0.45 (0.18–1.13)	0.089
	Juxta-vascular	Yes	0.41 (0.13–1.30)	0.131	0.41 (0.12–1.37)	0.149
AI 1	Location	Peripheral	Reference		Reference	
		Central	0.38 (0.19–0.76)	0.006	0.35 (0.18–0.71)	0.004
	Internal structure	Solid	Reference		Reference	
		GGN	0.52 (0.20–1.35)	0.18	0.53 (0.20–1.40)	0.2
	Atypical cyst	Yes	0.29 (0.05–1.55)	0.148	0.20 (0.04–1.12)	0.068
AI 2	Mass	Yes (≥ 3 cm)	0.27 (0.11–0.69)	0.006	0.24 (0.09–0.64)	0.004
	Juxta-pleural	Yes	0.70 (0.41–1.19)	0.182	0.86 (0.49–1.51)	0.609
	Multiple nodules	Yes (≥ 10)	0.66 (0.39–1.12)	0.125	0.54 (0.31–0.95)	0.033
	Atypical cyst	Yes	0.32 (0.07–1.46)	0.142	0.28 (0.06–1.41)	0.123

Univariate analysis used logistic regression, with Fisher’s exact test applied for variables with complete separation

AI 1 = Fujifilm Corporation; AI 2 = Plusman LLC

aOR: adjusted odds ratio, CI: confidence interval, OR: odds ratio. GGN: ground-glass nodule

## Discussion

In this comparative study of pulmonary nodule or mass detection on clinical CT scans, we demonstrated two principal findings. First, our results indicate that board-certified radiologists achieved higher detection sensitivity than the two commercially available AI systems. Second, this investigation identified a fundamental divergence in the types of features that limited performance: human detection was primarily influenced by intrinsic nodule or mass characteristics such as internal structure and proximity to anatomical features, whereas AI detection was more susceptible to case-level complexity, including the presence of multiple nodules or masses, and specific morphologies like very large or atypical cystic nodules or masses. These results provide crucial insights into the current capabilities and distinct limitations of widely used AI tools compared to human experts in a real-world clinical setting.

Our primary finding that board-certified radiologists demonstrated higher detection sensitivity than both commercial AI systems is noteworthy, as it diverges from trends reported in some recent large-scale analyses. Several systematic reviews and meta-analyses concluded that, on average, AI systems achieve higher pooled sensitivity than human radiologists [8, 12]. However, this discrepancy may be explained by the specific context of our study. A potential concern in retrospective reader studies is the “laboratory effect,” wherein human readers might adopt an artificially low decision threshold to maximize sensitivity, inevitably leading to excessive FPs [13]. Our data argue against this bias. The PPVs achieved by our radiologists (66.0% and 47.8%) were comparable to the 64% PPV reported for radiologists in a similar enriched cohort study by Huang et al. [14]. This suggests that our readers maintained a clinically relevant standard for positive calls and did not achieve high sensitivity merely through overdiagnosis. Instead, the superior sensitivity of human readers in our study must be interpreted in the context of the imaging protocol and the specific patient population. A crucial technical factor is the use of 5-mm slice thickness. Commercial AI algorithms are typically trained and optimized using high-resolution, thin-slice (e.g., 1–1.25 mm) screening datasets. When applied to 5-mm slice thickness clinical scans, the partial volume effect significantly degrades the contrast and edge definition of small nodules or GGNs. This technical “domain shift” from ideal training data likely contributed to the lower sensitivity of the AI systems. Furthermore, from a clinical perspective, routine diagnostic CTs frequently feature complex comorbidities such as consolidation, atelectasis, or scarring, which differ substantially from the clearer lung fields typical of screening cohorts. Experienced human radiologists excel at contextual interpretation, perceptually compensating for thicker slices and distinguishing true nodules from surrounding complex pathologies, whereas AI systems struggle with this real-world clinical complexity.

Our findings are particularly informative when compared with previous studies validating the same AI systems. For instance, Fukumoto et al. recently validated the performance of AI 1 (Fujifilm Corporation) on low-dose screening CTs in a Japanese cohort [9]. In their thin-slice screening dataset, AI 1 demonstrated robust sensitivity with a low FP rate. In contrast, when we applied the same AI to 5-mm thick routine clinical scans, its sensitivity was significantly compromised by case-level complexity (e.g., multiple nodules) and GGNs. This discrepancy highlights that AI performance optimized for screening does not seamlessly translate to routine clinical practice, highlighting the vulnerability of AI to “domain shifts” in image resolution and patient populations. Similarly, the specific design features of AI 2 (Plusman LLC), which applies a uniform 2-mm threshold without specialized algorithms for subsolid or atypical morphologies, may explain its distinct failure to detect very large masses (≥ 30 mm) and atypical cystic lesions, categorizing them instead as normal structures or consolidation. Furthermore, the difference in predefined detection thresholds between the two AI systems structurally influenced their comparative performance outcomes, particularly regarding FP rates and PPV. Because our reference standard was strictly aligned with AI 1’s thresholds (≥ 3 mm for solid, ≥ 5 mm for GGNs), AI 2’s default capability to detect smaller nodules (≥ 2 mm) inherently disadvantaged it in our evaluation framework. Any true nodules measuring between 2 mm and our reference thresholds that were detected by AI 2 were necessarily classified as FPs. This structural discrepancy likely inflated AI 2’s FP rate and disproportionately lowered its PPV compared to AI 1, highlighting the difficulty of comparing algorithms with varying default detection specifications.

A central finding of this study was the divergence in characteristics that challenged human readers versus AI systems. Our multivariate analysis revealed that Reader A’s detection was significantly affected by intrinsic nodule or mass features, including GGN structure (aOR: 0.21), central location (aOR: 0.40), proximity to vessels, and notably, the presence of consolidation (aOR: 0.07), which likely acted as a visual distractor. This aligns with well-documented “blind spots” in chest CT interpretation, where anatomical complexity and overlapping structures hinder human perception [15]. In contrast, Reader B, who achieved the highest sensitivity, showed no statistically significant limitations in the multivariate analysis, suggesting that these morphological challenges can be overcome with sufficient expertise. The AI systems, however, were negatively affected by different categories of features. AI 1 was uniquely challenged by central locations (aOR: 0.35; *p* = 0.004), a region notoriously complex because of hilar structures and dense vasculature. AI 2 was highly susceptible to case-level complexity and atypical morphologies, showing significantly lower sensitivity in cases with a high nodule burden (≥ 10 nodules; aOR: 0.54) and for large masses ≥ 30 mm (aOR: 0.24; *p* = 0.004). It also showed a similar trend of lower detection for atypical cysts (aOR: 0.28; *p* = 0.123). These specific failures likely reflect a “training data bias,” in which algorithms trained on screening datasets—typically featuring solitary, small, peripheral nodules—fail to recognize large, obvious tumors or complex lesions, instead categorizing them as organs, consolidation, or benign changes [15–17]. Overall, these findings suggest that AI limitations emerge in the context of anatomical complexity, high disease burden, and atypical presentations, areas in which the contextual understanding of human radiologists remains essential.

The broader implications of our findings—that experienced radiologists surpassed AI in sensitivity and that their weakness profiles are complementary—align with the current challenges in AI implementation. Consistent with recent literature, our data confirm that FP management and the risk of unnecessary workups remain major bottlenecks to clinical adoption [8, 12]. Consequently, the optimal role for current AI systems is likely not as autonomous readers, but as complementary tools deployed where their incremental sensitivity outweighs the FP cost. These findings have critical implications for the complementary use of AI in clinical practice. Since AI 1 shares similar blind spots with average radiologists (e.g., GGNs and a trend for central location), it may provide limited incremental value for these specific targets compared to AI 2, which demonstrated robustness to nodule texture. However, both AIs struggled with complex cases involving multiple nodules, and AI 2 missed large masses. Therefore, radiologists must remain vigilant for large, obvious masses and perform thorough checks in cases with high nodule counts, rather than relying solely on AI”safety nets.” A comprehensive workflow combining the radiologist’s ability to interpret complex pathology (like consolidation) with AI sensitivity to subtle nodules offers the most robust strategy. However, simply introducing AI as a second reader does not guarantee synergy; evidence indicates that suboptimal workflow design can cause the combined approach to underperform compared to either humans or AI alone [8, 18]. Therefore, future research must move beyond simple performance metrics and focus on implementation science. Specifically, future studies should: (i) report paired sensitivity-PPV endpoints on diverse, real-world datasets; (ii) quantify how case-level factors (e.g., multiple nodules) modulate performance; and (iii) prospectively test human–AI collaboration workflows with pre-specified triage rules optimized jointly for sensitivity and PPV [18–20]. Finally, as AI algorithms continue to evolve and potentially overcome current perceptual limitations, the core skill set required of radiologists is expected to shift. Rather than focusing primarily on exhaustive visual detection, future radiologists will need to excel in higher-order cognitive tasks, including the interpretation of imaging findings within the context of complex, patient-specific clinical information, guidance of personalized clinical management, and effective communication within multidisciplinary teams. In these domains, holistic clinical reasoning and human judgment remain irreplaceable.

Our study has some limitations. First, this was a retrospective, single-center study, which may limit generalizability. Second, the performance of human readers was based on only two board-certified radiologists, which may not be generalizable to all radiology specialists. As a next step, we plan to conduct a future reading experiment involving a larger pool of radiologists with varying levels of experience and subspecialties. Third, although the reference standard was rigorously established through independent review and consensus between two board-certified radiologists to mitigate single-reader bias, the inherent subjectivity of imaging interpretation means that some residual bias cannot be entirely eliminated. Fourth, as noted in the Discussion, differences in predefined detection thresholds between the two AI systems structurally influenced the comparative outcomes. Because the reference standard was aligned with AI 1 thresholds (≥ 3 mm for solid nodules and ≥ 5 mm for GGNs), AI 2, which is designed to detect smaller nodules (≥ 2 mm), was inherently disadvantaged. Nodules measuring between 2 mm and the reference thresholds, although correctly detected by AI 2, were classified as FPs, thereby limiting a fully equivalent comparison. Finally, the analysis was conducted using 5-mm slice thickness CT scans. Although this reflects routine clinical practice, it inherently limits the precise evaluation of internal characteristics, thereby precluding the subclassification of GGNs (e.g., distinguishing between pure GGN and part-solid), which requires thin-slice high-resolution CT [21, 22]. Consequently, we adopted a simplified classification of solid nodules versus GGNs.

In this study, participating board-certified radiologists demonstrated higher sensitivity and PPV than the evaluated commercial AI systems. We identified distinct performance limitations, with humans struggling with subtle intrinsic nodule or mass features and AI systems being challenged by case-level complexity and atypical morphologies. Therefore, current AI systems are best suited as complementary tools to support radiologists rather than as autonomous readers, provided that FPs are effectively managed.

## Supplementary Information

Below is the link to the electronic supplementary material.

Supplementary Material 1
